# Emerging roles of PGPR in fruit production systems: from growth promotion to system-level regulation, a review

**DOI:** 10.3389/fmicb.2026.1879927

**Published:** 2026-08-12

**Authors:** Mengqi Zhao, Ming Li, Biao Zhang

**Affiliations:** 1 College of Viticulture and Enology, Ningxia University, Yinchuan, China; 2 Ningxia Institute of Grape and Wine, Yinchuan, China; 3 Engineering Research Center of Grape and Wine, Ministry of Education, Yinchuan, China; 4 College of Life Sciences, Northwest University, Xi'an, China

**Keywords:** fruit production systems, induced systemic resistance, PGPR, rhizosphere microbiome, system-level regulation

## Abstract

With the growing importance of sustainable fruit production and the increasing demand for reduced reliance on chemical fertilizers and pesticides, plant growth-promoting rhizobacteria (PGPR) have attracted considerable attention as promising biological resources in orchard systems. However, current research is mainly constrained by an overreliance on single-trait interpretations of PGPR function, which cannot fully explain their performance under the complex, heterogeneous, and long-term conditions of perennial fruit production. This review provides a systematic overview of the emerging roles of PGPR in fruit production systems from a system-level perspective. Drawing on representative recent studies, this review first summarizes the shift from classical growth-promotion functions to integrated system-level regulation. It then discusses current research progress from three interrelated dimensions: rhizosphere engineering and synthetic microbiome assembly, physiological regulation of plant stress tolerance, and molecular signaling associated with induced systemic resistance. Particular emphasis is placed on key mechanisms including extracellular polymeric substance secretion, ACC deaminase activity, antioxidant regulation, hormone crosstalk, and defense priming, as well as on the role of multi-strain consortia and multi-omics approaches in linking microbial traits with host responses and rhizosphere ecological processes. This review shows that PGPR in fruit production systems function not only as direct growth promoters but also as regulators of interconnected plant–soil-microbiome processes involved in nutrient acquisition, stress adaptation, disease resistance, and fruit quality formation. Nevertheless, important limitations remain, including inconsistent field performance, weak colonization persistence, insufficient fruit tree-specific evidence, formulation difficulties, and biosafety concerns. Future research should focus on host- and environment-specific inoculant design, mechanistic validation under orchard conditions, integration of multi-omics with long-term field evaluation, and the development of persistence-oriented and safety-assessed application strategies, thereby providing a stronger theoretical and practical foundation for sustainable orchard management.

## 1 Introduction

Fruit production systems are under increasing pressure to maintain high yield and fruit quality while reducing dependence on chemical fertilizers and pesticides (De Mastro et al., 2025). Climate change (Cosmulescu, 2026), soil degradation (Huang et al., 2025; Xie et al., 2022), and rising disease pressure (Bacelar et al., 2024; He et al., 2025) further complicate orchard management, particularly in perennial systems, where root longevity, seasonal fluctuations, and accumulated soil effects strongly influence long-term productivity (Chen, 2025). Compared with annual crops, fruit trees depend on stable belowground ecological processes more strongly to sustain productivity, fruit quality, and resilience over time.

In this context, plant growth-promoting rhizobacteria (PGPR) have gradually emerged as a potential biological tool for enhancing the sustainability of fruit production. In fruit crops, PGPR can stimulate root development, improve nutrient uptake, alleviate abiotic stresses such as salinity and drought, and enhance resistance to pathogens. These beneficial effects are highly relevant to orchard systems, where excessive chemical inputs can degrade soil health, disrupt beneficial microbial communities, and reduce the long-term sustainability of production. As a result, PGPR-based approaches are increasingly viewed as important components of low-input and environmentally responsible fruit cultivation strategies.

However, accumulating evidence suggests that a trait-based understanding alone is insufficient to explain the performance of PGPR in field-grown fruit trees (Chang et al., 2025; Nicotra et al., 2026). Many early studies focused on individual microbial functions, such as phosphate solubilization (Zhou et al., 2025), phytohormone production (Yang et al., 2025), nitrogen fixation (Pelagio-Flores et al., 2025), and pathogen antagonism (Mazuecos-Aguilera et al., 2025). Although these traits remain important, they do not fully explain PGPR performance in the complex and heterogeneous conditions of orchard ecosystems. In perennial fruit systems, the efficacy of PGPR inoculation is shaped not only by the functional capacity of the introduced strain, but also by the physicochemical properties of the rhizosphere, host physiological status, indigenous microbial communities, seasonal variation, and long-term plant–soil feedbacks. Therefore, PGPR-mediated benefits should not be interpreted simply as the sum of several isolated microbial functions.

A system-level perspective is particularly important for fruit crops because orchard performance emerges from interactions across multiple biological scales. Rather than acting through isolated pathways, PGPR often regulate interconnected processes that span the rhizosphere environment, plant physiological homeostasis, and molecular defense signaling. At the rhizosphere level, PGPR can modify the physicochemical properties of the root-associated microenvironment, improve soil structure, buffer ionic and water stress, and influence microbiome assembly (Shi et al., 2026; Lim and Kim, 2013). At the plant physiological level, PGPR can reduce excessive stress ethylene through ACC deaminase activity, stimulate antioxidant enzyme systems to control reactive oxygen species, and modulate endogenous hormone balance, thereby helping plants maintain growth and cellular homeostasis under adverse conditions (Yang et al., 2026). At the molecular level, PGPR or their metabolites can act as elicitors that are perceived by plant receptors, triggering signaling cascades involving salicylic acid (SA), jasmonic acid (JA), and ethylene (ET), and ultimately promoting defense priming and induced systemic resistance (Kesavardhini et al., 2025). These layers are interconnected rather than independent: rhizosphere modification affects microbial colonization and metabolite exchange, physiological stabilization determines host responsiveness, and molecular signaling coordinates downstream adaptive outcomes (Choudhary et al., 2007; Zaib et al., 2023; Devin et al., 2023). Together, these findings support the view that PGPR regulate integrated plant–soil-microbiome systems.

At the same time, advances in transcriptomics, metabolomics, and microbiome profiling have fundamentally reshaped how PGPR functions are understood. Traditional cultivation-based experiments and phenotypic observations often describe visible outcomes such as improved biomass, nutrient content, or disease suppression, but they provide limited insight into how these outcomes are generated under complex field conditions. Multi-omics approaches help to reveal deeper regulatory processes, including shifts in host gene expression, metabolic reprogramming, redox regulation, hormone signaling, and rhizosphere community assembly following PGPR inoculation (Yuan et al., 2025; Muhammad et al., 2023). These approaches increasingly link microbial activity with host responses and environmental context, moving the field beyond descriptive observations toward a more mechanistic and integrative understanding. For fruit production systems, this shift is especially valuable because orchard performance depends on the long-term coordination of plant physiology, soil conditions, and microbial ecology rather than on a single short-term growth response.

Furthermore, the adoption of a system-level framework is necessitated by the fact that microbial inoculation in orchard systems rarely occurs in a biological vacuum (Liu et al., 2022). Introduced PGPR must establish themselves within pre-existing rhizosphere communities, compete or cooperate with resident microorganisms, and function under heterogeneous soil and climatic conditions. In some cases, the most important contribution of a PGPR strain may not be its direct effect on the host alone, but its ability to restructure rhizosphere interactions, recruit beneficial indigenous microbes, or stabilize a functionally complementary microbial consortium (Zhang et al., 2022). This possibility has stimulated growing interest in synthetic microbiome design, in which strains with distinct but complementary functions are intentionally combined to generate more robust and predictable outcomes than single-strain inoculants. For perennial fruit crops, where long-term stability and ecological compatibility are essential, such community-based approaches may provide a more realistic path toward durable PGPR application.

**Figure 1:**
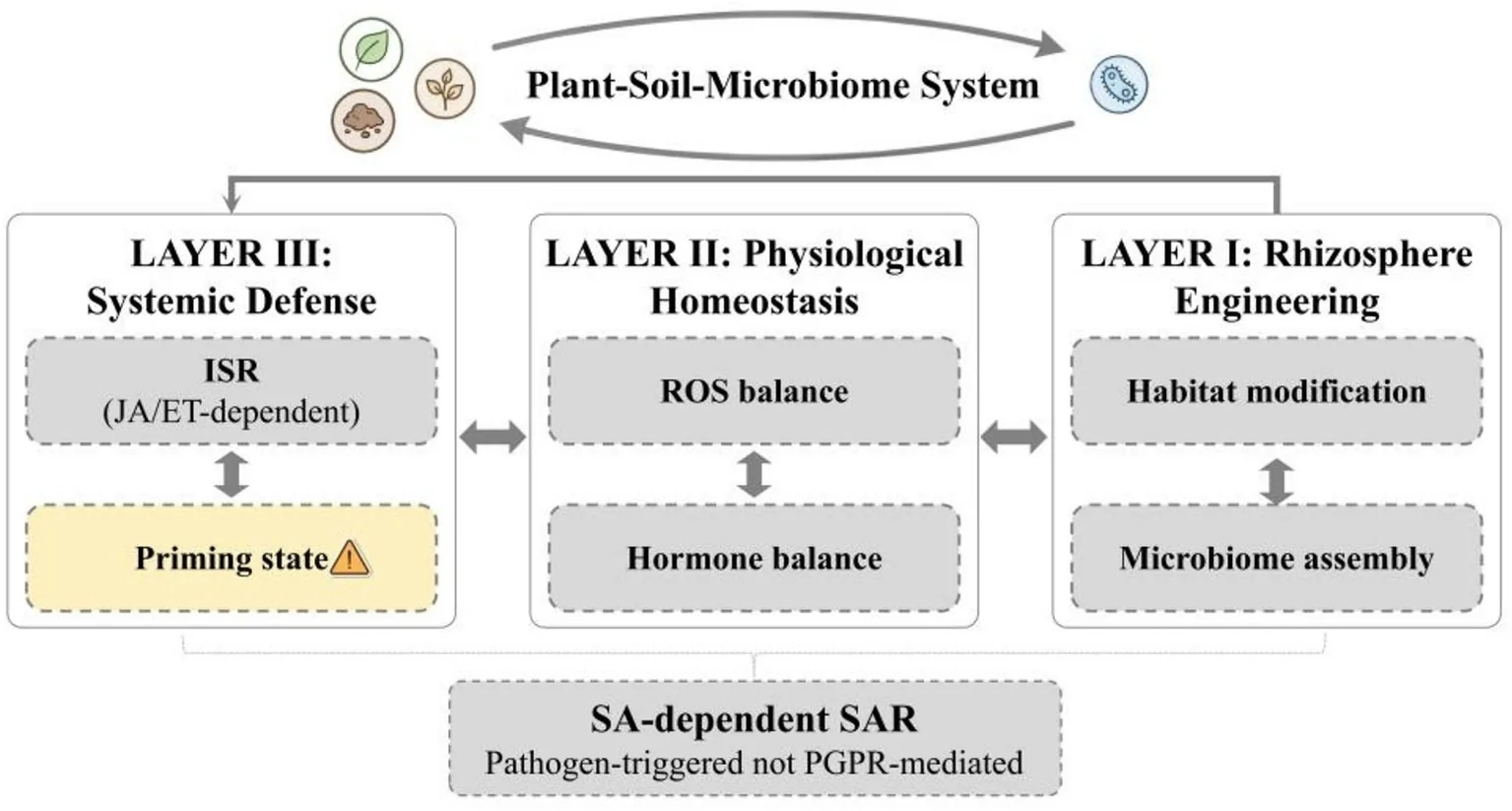
Multi-level framework diagram for PGPR-mediated regulation in perennial fruit production systems.

Given the difficulty of translating community-level benefits into stable field outcomes across diverse fruit production systems, a system-level mechanistic framework is needed. In this context, the present review summarizes recent advances in PGPR research in fruit production systems from a system-level perspective. Guided by the framework presented in Figure 1, this review focuses on three interrelated mechanistic layers: (i) rhizosphere engineering and synthetic microbiome assembly, (ii) physiological regulation of plant stress tolerance, and (iii) molecular signaling and induced systemic resistance. By organizing the literature according to these interconnected levels, the review aims to clarify how PGPR function in perennial orchard environments and why their effects cannot be adequately explained by isolated beneficial traits alone. Finally, future perspectives are briefly outlined to highlight key priorities for orchard-specific validation, stable colonization, and ecologically robust PGPR application in sustainable fruit production.

## 2 Mechanistic basis of PGPR-mediated regulation in fruit production systems

PGPR-mediated regulation in fruit production systems can be understood as a multi-layered process operating across the rhizosphere, plant physiology, and molecular signaling networks. Importantly, the validity of each mechanistic claim depends on the level of evidence: mechanisms supported only by annual crop or model plant studies are treated here as hypotheses requiring validation in perennial fruit systems, rather than as established facts. As shown in Figure 1, the following sections discuss these three mechanistic levels in sequence, with explicit evidence grading: mechanisms with direct fruit tree validation are presented as established; mechanisms supported only by studies on annual crops or model plants are explicitly labeled as conserved physiological principles requiring perennial-specific verification.

### 2.1 Rhizosphere engineering and synthetic microbiome assembly

#### 2.1.1 Environmental adaptation: the first constraint for PGPR efficacy in orchards

In perennial orchard systems, root performance is not determined solely by plant intrinsic traits, but also by the physical, chemical, and biological properties of the root-associated microenvironment. These attributes are continuously shaped by long-term root–soil interactions, seasonal changes, irrigation regimes, and accumulated management practices (Tong et al., 2026; Tanur Erkoyuncu et al., 2026; Verma et al., 2024).

Early studies on PGPR in agricultural and horticultural systems mainly emphasized individual microbial traits directly associated with plant growth promotion (Chang et al., 2025). Since the concept of PGPR was formally introduced by Kloepper and Schroth in 1978, numerous bacterial strains have been characterized for biological nitrogen fixation, phosphate solubilization, phytohormone production, and siderophore secretion (Pelagio-Flores et al., 2025; Hu et al., 2026). These trait-based studies, conducted mainly in annual crops or under controlled conditions, provided an important foundation for microbial inoculant development but do not constitute direct evidence for orchard systems.

However, the assumption that these individual traits predict field performance in orchard systems has been increasingly challenged. Evidence from annual crops indicates that strains possessing only one or a few direct growth-promoting traits often show limited or unstable performance under field conditions (Hu et al., 2026; Wang et al., 2026).

**Figure 2:**
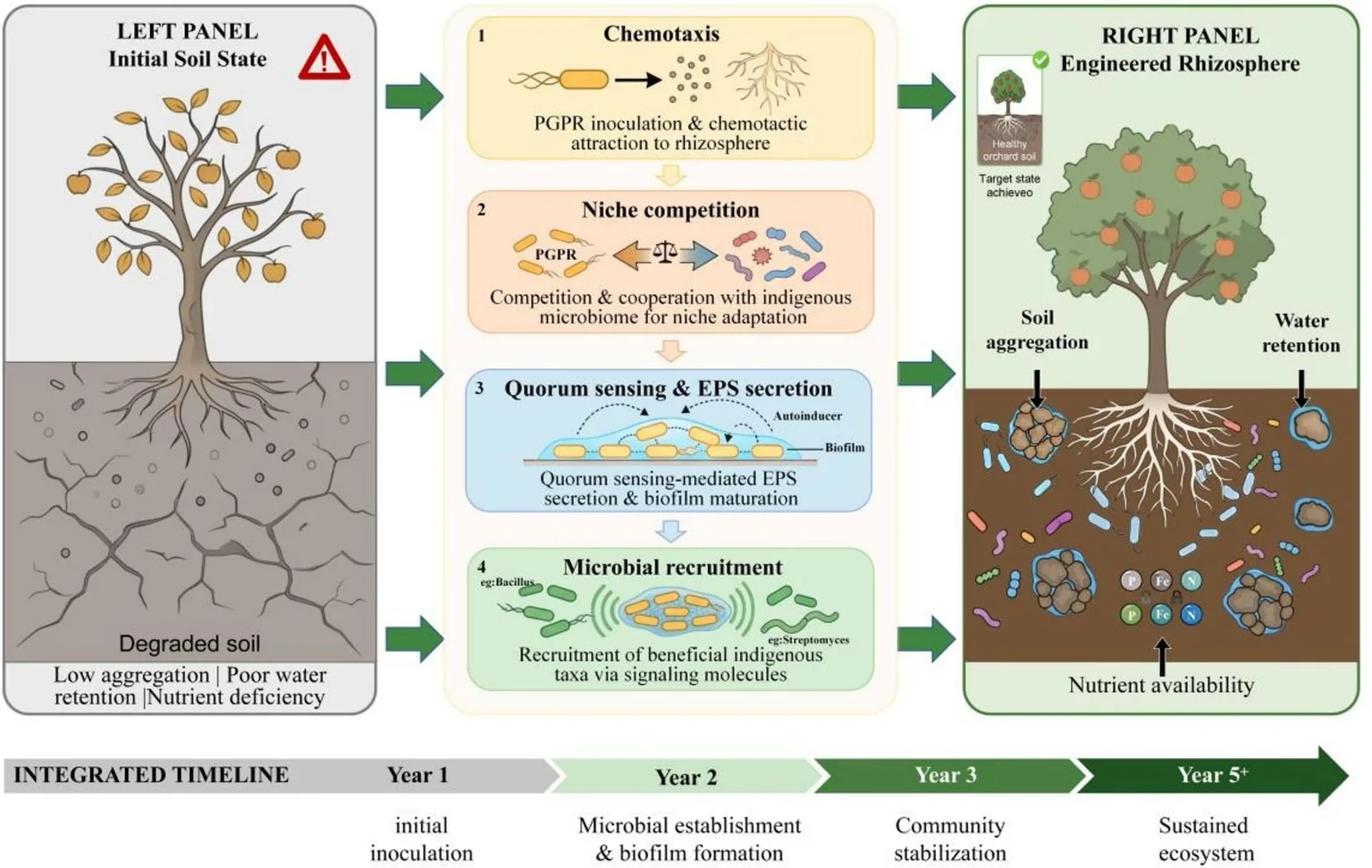
Rhizosphere engineering and microbiome assembly.

In fruit production systems, where soil pH, salinity, moisture status, organic matter content, and indigenous microbial communities vary substantially across sites and seasons, microbial inoculants must establish, persist, and function within a complex rhizosphere environment (Figure 2) (Hu et al., 2026; Wang et al., 2026). This environmental filtering effect has been directly validated in perennial crops: Palladino et al. demonstrated that altitude and soil type significantly filtered rhizosphere microbial populations carrying plant-beneficial genes in grapevine, with functional markers such as nifH and acdS enriched in healthy vineyards but reduced in degraded soils (Ferreira et al., 2026). This evidence from a perennial fruit crop suggests that PGPR efficacy depends not only on the inoculated strain itself but also on whether the surrounding soil can support its colonization, metabolic activity, and interaction with resident microbiota. Accordingly, modern inoculant design for orchard systems should prioritize environment-adapted strain selection for ecological fitness under orchard-specific constraints, including high pH, low iron availability, phosphorus limitation, salinity, and intermittent drought (Wang et al., 2026; Ferreira et al., 2026; Díaz-Rodríguez et al., 2025).

#### 2.1.2 From single strains to complementary consortia: ecological principles

A further dimension of rhizosphere engineering is microbiome assembly. Introduced PGPR do not act in isolation; once applied to the soil or root system, they enter a dense ecological network composed of indigenous bacteria, fungi, root exudates, and soil metabolites (Arnault et al., 2024). Their effectiveness therefore depends partly on how they interact with resident communities through competition, facilitation, signaling, and niche modification (Garrido-Sanz et al., 2023).

In some cases, the most important contribution of an inoculated strain may not be its direct action on the host alone, but its capacity to recruit, enrich, or stabilize other beneficial microorganisms already present in the rhizosphere (Nicotra et al., 2026). For instance, seed inoculation with synthetic communities (SynComs) on common bean demonstrated that engineered microbiota contribute up to 80% of the seedling microbiome and modify subsequent rhizosphere assembly through priority effects, even when SynCom members constitute less than 0.2% of the rhizosphere community (Nicotra et al., 2026). This annual-crop evidence illustrates the principle of microbiome steering. However, whether priority effects operate similarly in established orchard soils, where resident communities are already well developed and multi-season persistence is required, remains to be tested in perennial systems.

This insight has led to growing interest in synthetic microbiome assembly as an extension of traditional PGPR application. Rather than relying on a single strain expected to perform multiple roles simultaneously, synthetic microbiome approaches intentionally combine strains with distinct but complementary traits to generate more robust and predictable outcomes (Zhang et al., 2026).

In solanaceous vegetables, Xu et al. engineered a four-member pepper SynCom where *B. subtilis* handled biofilm/ISR, Trichoderma spp. managed nutrient/hormone functions, and Aspergillus sp. conducted P-solubilization, yielding synergistic disease suppression and growth promotion (Xu et al., 2025). This annual-crop evidence demonstrates functional partitioning but provides no direct data on multi-season persistence in woody root systems, compatibility with established orchard microbiomes, or synchronization with perennial growth cycles.

Recent studies in annual crops reveal that high-relatedness (HR) strains exhibit substantial metabolic niche overlap, leading to intense resource competition and suppressed plant growth-promoting functions (He et al., 2025; Jia et al., 2026). Moderate-relatedness (MR) consortia with complementary niches demonstrate superior performance (He et al., 2025; Benmrid et al., 2025). Although derived from annual crops, these findings generate a testable hypothesis for perennial systems: in orchards, where introduced strains must coexist with well-established indigenous microbiomes over multiple growing seasons, metabolic niche overlap may be amplified by priority effects and competitive exclusion. However, the design principles for achieving niche complementarity in established orchard microbiomes remain empirically undefined.

#### 2.1.3 Rhizosphere modification: EPS, biofilm, and root habitat improvement

Rhizosphere engineering refers to the capacity of PGPR to modify the root-associated environment in ways that improve root function and enhance stress-buffering (Jia et al., 2026). In natural and managed systems, beneficial microorganisms can influence soil aggregation, nutrient accessibility, microsite stability, and the abundance of beneficial microbial partners in the root zone (Pradhan et al., 2025; González-Cruz et al., 2025). These effects are particularly significant in fruit trees because root systems remain active in the same soil for multiple years, making them highly sensitive to cumulative changes in rhizosphere structure and microbial composition (Yaseen, 2026). By improving the quality of the root habitat, PGPR can indirectly enhance the efficiency of water and nutrient acquisition, support continuous root activity, and create a more favorable context for long-term plant performance (Verbon and Liberman, 2016; Grover et al., 2021; Zuluaga et al., 2026).

EPS-mediated stress buffering has been characterized primarily in annual crops and controlled conditions: under saline conditions, EPS can adsorb or chelate sodium ions, limiting their movement toward the root surface and helping maintain ionic homeostasis (Giannelli et al., 2023); EPS-rich biofilms protect roots and microbes from osmotic fluctuations (Shi et al., 2026); under drought, EPS contributes to soil aggregate stability and water retention in the root zone (Parastesh et al., 2025). These mechanisms are physiologically plausible in fruit trees but remain poorly validated under orchard conditions. In particular, the extent to which EPS-mediated Na^+^ chelation alters K^+^/Na^+^ ratios in mature roots, and the durability of biofilms under seasonal freeze–thaw cycles, remain unknown.

Through these functions, PGPR do not merely “promote growth” in a narrow sense. Although direct evidence from perennial fruit systems remains limited, Zeng et al. demonstrated that PGPR volatile organic compounds enhanced sweet cherry root system development, including increased root length and branching (Zeng et al., 2025). As shown in Table 1, this finding indicates that PGPR-mediated rhizosphere improvement can enhance root performance in fruit production systems, where long-term absorptive capacity is critical for yield stability. However, whether these root architectural changes result from EPS-mediated habitat improvement, direct hormone signaling, or microbiome restructuring remains unresolved in perennial crops.

**Table 1:** Representative PGPR with direct evidence from fruit production systems.

PGPR taxon/strain	Fruit crop	Verified functions	Primary mechanisms	Evidence type	Application considerations/limitations	References
*Pseudomonas putida KT2440*	Citrus	Heat/high light stress tolerance; oxidative stress alleviation	Enhancement of catalase and related antioxidant enzymes; mitigation of membrane oxidative damage under high temperature and intense light	Direct: potted citrus under controlled high-temperature and high-light stress; antioxidant enzyme activities and membrane stability measured	Exemplary direct fruit tree case for abiotic stress sections; controlled conditions limit generalization to open orchard environments	Terán et al. (2025)
*Pseudomonas* spp. *(ISR-related strains)*	Citrus	ISR	Priming of defense-related genes; regulation of SA/JA/ET-associated signaling pathways; enhancement of antioxidant capacity	Partially direct: citrus-associated soils and other crop-related systems; strain-host specificity demonstrated in citrus rhizosphere	Mechanistically significant for fruit trees, but strain-host specificity should be emphasized; transferable to other perennial systems with caution	Mazuecos-Aguilera et al. (2025)
*Drought-tolerant PGPR consortia (BC3, BC4)*	Grapevine (*Vitis vinifera*)	Drought tolerance; growth promotion; photosynthetic maintenance	IAA and EPS production under osmotic stress; biofilm formation; enhanced root biomass; maintenance of stomatal conductance under water deficit	Direct: one-year-old grapevines subjected to progressive drought stress in pot trial; BC3 increased shoot length by 35.5%, BC4 by 26.5%	Consortium-specific effects: BC3 enhanced early growth under well-watered conditions, BC4 conferred greater benefits under water deficit; field validation needed	Canavera et al. (2026)
*Marine PGPR consortia (A. aquariorum, B. methylotrophicus, B. aryabhattai)*	Grapevine (*Vitis vinifera* cv. Antão Vaz)	Heatwave stress tolerance; photoprotection; membrane stability	Enhanced photoprotection capability; lower dissipation energy flux; improved light-harvesting via increased reaction centre availability; osmoprotectant promotion; antioxidant mechanisms reducing lipid peroxidation	Direct: grapevines exposed to heatwave conditions; photochemical traits, pigment profiles, and oxidative stress biomarkers analyzed	Consortium 1 conferred stress avoidance, Consortium 2 conferred increased resistance; effectiveness differed significantly between consortia despite similar in vitro potential	Carreiras et al. (2023)
*Pseudomonas* spp. *(C11, C24, C26, C6 isolates)*	Citrus (Citrus macrophylla rootstock)	Growth promotion; seed germination; root system proliferation	IAA production; phosphate solubilization; siderophore synthesis; antibiotic production inhibiting pathogenic fungi and bacteria	Direct: fluorescent Pseudomonas isolated from citrus rhizosphere; tested on *C. macrophylla* rootstock in greenhouse conditions; significant improvement in plant height, collar diameter, and root length	Greenhouse validation only; field performance in mature citrus orchards remains untested; potential biofertilizer alternative for citrus rootstock production	Qessaoui (2020)
*Multi-strain PGPR consortia*	Strawberry (Fragaria × ananassa cv. Chandler)	Growth promotion; yield increase; fruit quality improvement; rhizosphere stochiometry enhancement	Functional complementarity; enhanced biofilm formation; improved rhizosphere colonization; coordinated nutrient and defense regulation; increased acid/alkaline phosphatase and dehydrogenase enzyme activity	Direct: strawberry cv. Chandler grown on solarized organic soils; 250 g PGPR consortium significantly improved vegetative growth, fruit number, cumulative yield, and fruit quality traits	Soil solarization synergized with PGPR; effects on calcareous soils of Shiwalik foothills; cultivar and soil-type dependency should be considered	Kumar et al. (2020)
*PGPR consortia (P. fluorescence, B. subtilis, A. chroococcum, K-mobilizing bacteria, AM fungi)*	Strawberry (Fragaria × ananassa)	Growth promotion; fruit yield; fruit quality; runner production	Nutrient mobilization (N, P, K, Mg); enhanced leaf nutrient concentration; improved rhizosphere microbial count; increased DOP and ΣDOP indexes	Direct: field-grown strawberry over multiple growing seasons (2019–2020, 2020–2021); PGPR improved total yield, marketable fruit weight, and fruit number	Effects cultivar-dependent; PGPR application during flower induction increased total yield and fruit number in *F. vesca* and F. × ananassa; sucrose content altered in cultivar-specific manner	Bhat et al. (2025) and Kurokura et al. (2017)
*PGPR volatile organic compounds (VOCs)*	Sweet cherry (*Prunus avium*)	Root system development; increased root length and branching	Volatile organic compound-mediated root growth stimulation; enhanced absorptive capacity	Direct: sweet cherry root system development measured; increased root length and branching documented	Direct orchard evidence confirms PGPR-mediated rhizosphere improvement can translate into sustained root performance; whether effects result from EPS, hormone signaling, or microbiome restructuring remains unresolved	Zeng et al. (2025)
*Microbial biofertilizers (multiple strains)*	Apple (*Malus domestica* cv. Gala)	Growth promotion; yield improvement; chemical fertilizer reduction	Biological nitrogen fixation; phosphate solubilization; phytohormone production; enhanced nutrient uptake	Direct: multiyear field evaluation in Gala apple trees; effects markedly dependent on strain, rootstock, cultivar., and evaluation metrics	Strong host and environmental dependence demonstrated; exemplifies field inconsistency challenge; multi-season persistence not fully characterized	Ferreira et al. (2026)
*Multi-strain PGPR consortia*	Tomato, grape, apple, and other horticultural systems discussed in reviews	Simultaneous improvement of growth, stress resistance, and fruit quality	Functional complementarity, enhanced biofilm formation, improved rhizosphere colonization, coordinated nutrient and defense regulation	Inconsistent evidence: partially direct, partially transferable	Currently the most relevant research direction, but effects are highly dependent on niche compatibility, orchard environment, and formulation quality	Wang et al. (2025) and Sarver et al. (2025)

#### 2.1.4 Downstream effects: root architecture, tree vigor, and fruit quality

At the root level, PGPR have been shown to increase primary root thickness and fine root abundance (Verbon and Liberman, 2016), thereby improving root architecture (Grover et al., 2021) and absorptive capacity (Zuluaga et al., 2026), which are associated with greater plant vigor including plant height, canopy development, leaf number, and chlorophyll content (Melini et al., 2023).

Direct evidence from perennial fruit trees supports these root architectural responses (Table 1). Cano-Castro et al. demonstrated that soil inoculation with *Bacillus velezensis* significantly improved both shoot and root system biomass in grapefruit seedlings, with the combination of PGPR and inorganic fertilization surpassing both negative and positive controls (Cano-Castro et al., 2024). This evidence indicates that PGPR-mediated rhizosphere improvement can enhance root performance in fruit production systems, where long-term absorptive capacity is critical for yield stability. By contrast, comparable studies in annual crops such as tomato provide mechanistic support but do not address the multi-season persistence required in orchard systems.

### 2.2 Physiological regulation of plant stress tolerance

In addition to their direct growth-promoting effects, PGPR enhance fruit production systems through an equally critical mechanism: their capacity to regulate plant physiological homeostasis under stress. In perennial fruit trees, stress tolerance is not determined solely by the avoidance of external damage, but also by the ability of plants to maintain internal metabolic balance when repeatedly exposed to salinity, drought, high temperature, excessive radiation, nutrient limitation, and soil-borne pathogens across multiple growing seasons (Yang et al., 2026; Kesavardhini et al., 2025). Under such conditions, the value of PGPR extends beyond growth promotion under favorable environments; more importantly, these microorganisms help plants preserve cellular integrity, redox balance, osmotic adjustment, and hormonal coordination under suboptimal orchard conditions (Yang et al., 2026; Kesavardhini et al., 2025; Grover et al., 2021).

Rather than simply stimulating biomass accumulation, beneficial rhizobacteria can modulate stress-associated metabolic pathways, reduce physiological damage, and stabilize plant function during prolonged or recurrent environmental stress (Ferrante et al., 2024; Sun et al., 2024). This perspective is particularly relevant for fruit trees because perennial crops experience cumulative stress exposure over time, and relatively small improvements in physiological regulation may translate into major differences in long-term vigor, fruit set, and yield stability.

#### 2.2.1 Redox balance, membrane stability, and osmotic regulation

**Figure 3:**
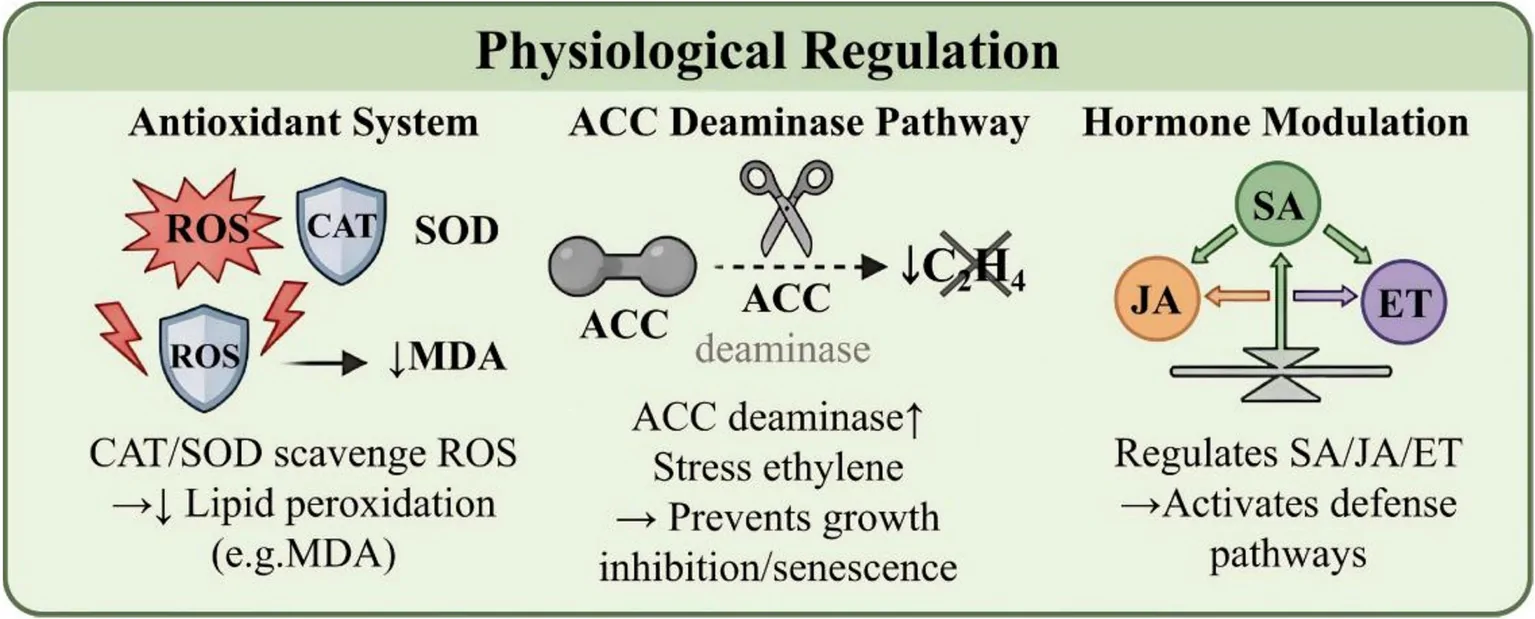
Physiological regulation mediated by PGPR: antioxidant responses, ACC deaminase activity, and hormone-related pathways.

PGPR-mediated physiological regulation contributes to plant stress tolerance not only through growth promotion, but also through the maintenance of redox balance, membrane stability, osmotic adjustment, and nutrient homeostasis (Figure 3). Under environmental stress, excessive accumulation of reactive oxygen species (ROS), including superoxide radicals, hydrogen peroxide, and hydroxyl radicals, can damage membranes, proteins, nucleic acids, and photosynthetic structures (Chen et al., 2026; Haider et al., 2026; Kang et al., 2025). In annual crops, PGPR inoculation has repeatedly been shown to stimulate antioxidant enzymes such as superoxide dismutase (SOD), catalase (CAT), ascorbate peroxidase (APX), and glutathione reductase (GR), thereby improving ROS detoxification and limiting oxidative injury (Yang et al., 2026; Haider et al., 2026). Reductions in malondialdehyde (MDA) have also been widely reported, indicating improved membrane stability under stress (Chen et al., 2026; Haider et al., 2026). These transferable findings support a conserved physiological principle: PGPR-mediated stress tolerance is often associated with redox homeostasis, although its magnitude and agronomic relevance in perennial fruit trees remain unclear.

Fruit tree-specific evidence provides more direct, but still limited, support for this mechanism. *Pseudomonas putida* KT2440 enhanced catalase-related antioxidant activity in citrus exposed to high temperature and intense light, alleviating oxidative damage under orchard-relevant conditions (Terán et al., 2025). However, some field studies in grapevine and apple have reported negligible changes in SOD or CAT activity despite improved growth performance (Mashabela et al., 2022). These observations suggest that antioxidant upregulation is not a universal response in orchard systems and may depend on stress type, intensity, phenological stage, and experimental timescale. Whether repeated or prolonged activation of antioxidant systems imposes metabolic trade-offs in long-lived perennial crops also remains unclear.

Beyond redox regulation, PGPR can support osmotic adjustment and ionic balance under drought and salinity stress. Under such conditions, plants must maintain cell turgor, water uptake, and nutrient balance despite declining water potential and increasing ionic toxicity. PGPR can facilitate these processes through improved root function, nutrient mobilization, and regulation of metabolic and ionic homeostasis (Dong et al., 2024; Kang et al., 2025). For example, some strains help maintain Na+/K + balance, thereby reducing sodium toxicity while preserving potassium-dependent cellular processes (Dong et al., 2024; Kang et al., 2025). Others improve phosphorus solubilization, micronutrient availability, or iron acquisition, helping to relieve stress-associated nutritional constraints and sustain metabolic function (Weselowski et al., 2016; Liu et al., 2017). Together, these findings provide a useful mechanistic framework, but perennial-specific factors such as deep root architecture, seasonal carbohydrate reserves, and multiyear stress exposure still require direct validation in mature fruit trees.

#### 2.2.2 Hormonal coordination and the ACC deaminase pathway

As shown in Figure 3, ACC deaminase-mediated ethylene reduction is one of the most widely validated physiological mechanisms—in annual crops. Many PGPR possess ACC deaminase, an enzyme that degrades 1-aminocyclopropane-1-carboxylate (ACC), the immediate precursor of ethylene, thereby lowering stress-induced ethylene accumulation in plant tissues (Pan et al., 2025; Ning et al., 2024). Through this mechanism, PGPR can prevent the overactivation of ethylene-dependent growth inhibition and help roots maintain elongation and absorptive function under salinity, alkalinity, and drought stress (Ferrante et al., 2024; Sun et al., 2024). E*nterobacter hormaechei*, E. roggenkampii, and *E. asburiae* have been identified as representative ACC deaminase-producing strains in tomato, sugarcane, and cucumber (Pan et al., 2025; Guo et al., 2020; Ning et al., 2024).

This transferable evidence demonstrates a conserved enzymatic function but provides no direct evidence for the following processes in orchard systems (Table 2): (i) ACC deaminase expression stability in orchard soils over multiple seasons, (ii) ethylene signaling dynamics in woody perennial roots with secondary growth, or (iii) dose–response relationships in mature fruit trees with large root biomass.

**Table 2:** Representative PGPR with indirect/transferable evidence (non-fruit crop or model plant studies).

PGPR taxon/strain	Fruit crop	Verified functions	Primary mechanisms	Evidence type	Application considerations/limitations	References
*Pseudomonas fluorescens*	Populus	Growth promotion; nutrient acquisition; induced resistance	Root growth stimulation, nutrient mobilization, antimicrobial metabolite production, host defense response activation	Indirect/transferable: Populus-associated strains; extensively studied in rhizosphere systems; mechanistic principles likely conserved in fruit trees	High functional diversity, but performance is context-dependent; generalization to orchard soils is not validated without empirical testing.	Timm et al. (2015)
⚠*Pseudomonas aeruginosa HG28-5*	Tomato	Salt stress alleviation; growth promotion	K^+^/Na^+^ homeostasis regulation, ABA-related responses, antimicrobial activity	Mechanistic reference only: tomato under salt stress; physiological mechanisms likely transferable but not fruit tree-specific	Potential biosafety concerns; unsuitable as a model application strain in agricultural recommendation sections	Dong et al. (2024)
⚠*Bacillus cereus DW019*	Cherry tomato	Growth promotion and stress resistance	Extracellular enzyme production, phytohormone-related effects, stress response pathway induction	Indirect/transferable: cherry tomato (annual crop); rhizospheric microbial community modulation demonstrated	Some *B. cereus* strains raise biosafety concerns; strain-level identification is required	Dong et al. (2023)
*Bacillus pumilus DZQ7*	Tobacco	Biological control; ISR-related protection	Antibiotic production, fungal germination inhibition, PR-related response induction	Mechanistic reference only: tobacco rhizosphere/soil-borne disease context; defense mechanisms demonstrated in annual crop	Can be cited as disease resistance example, but not as direct fruit tree application case	Hou et al. (2015)
*Paenibacillus polymyxa*	Rhizosphere of diverse crops	Growth promotion; nutrient mobilization; disease suppression	Antimicrobial peptide production, phosphorus solubilization, plant immunity enhancement	Mechanistic reference only: tobacco rhizosphere/soil-borne disease context; defense mechanisms demonstrated in annual crop	Promising multifunctional PGPR; field stability in orchards remains to be validated	Weselowski et al., (2016)
*Brevibacillus iranensis YZ29*	Peanut	Iron/phosphorus nutrition improvement; growth promotion	Enhanced nutrient solubilization, increased growth-related metabolite production, micronutrient uptake enhancement	Mechanistic reference only: peanut in calcareous soils; nutrient mobilization mechanisms characterized	More suitable for supporting nutrient mobilization mechanism discussions rather than fruit tree-related claims	Liu et al., (2017)
*Enterobacter hormaechei P03*	Tomato	Salt tolerance; growth promotion	ACC deaminase activity, IAA production, phosphorus solubilization capacity	tomato under saline conditions; ACC deaminase-mediated stress alleviation demonstrated	Suitable for ACC deaminase sections; biosafety and persistence assessments are required for orchard use	Pan et al. (2025)
*Enterobacter roggenkampii ED5*	Sugarcane/Cucumber	Drought/heat stress tolerance; ISR-related effects	IAA and siderophore synthesis, systemic resistance induction, stress mitigation	Mechanistic reference only: reported in sugarcane/cucumber; nitrogen-fixing endophytic bacterium	Taxon may contribute to mechanistic discussions but is not suitable as exemplary case for fruit tree applications	Guo et al. (2020)
*Bacillus licheniformis K11*	Pepper	Drought tolerance; improved nutrient availability	Enhanced enzyme production, improved drought resistance, increased nutrient accessibility	Indirect/transferable: pepper and droughtland-associated systems; drought resistance mechanisms characterized	Good supporting evidence for drought resistance mechanisms, but lacking direct fruit tree evidence	Lim and Kim (2013)
*Azospirillum brasilense and related species*	Cereals and field crops	Root growth and nutrient use efficiency improvement	Nitrogen fixation, IAA/cytokinin-related effects, root development stimulation	Indirect/background: cereals and field crops; classical PGPR reference	Can be cited as classical PGPR reference, but weak specificity for fruit production processes described in current manuscript	Tarrand et al. (1978) and Ayyaz et al. (2016)

This mechanism may be especially important in orchard crops, where root activity must be maintained over long periods in relatively stable soil environments.

Hormonal coordination represents another core component of physiological regulation. PGPR can influence endogenous plant hormone balance through the production or modulation of IAA, cytokinins, ABA-related responses, and ethylene-associated pathways (Tsukanova et al., 2017). Unlike the signaling-level discussion developed in Mechanism III, the physiological significance of hormone regulation at this stage lies in its contribution to whole-plant functional stability. For example, ABA-related responses influence stomatal behavior (Bouremani et al., 2023) and water conservation, auxin affects root growth and branching, and ethylene modulation determines whether plants maintain active growth or shift toward stress-induced senescence (Jamil et al., 2022).

These findings from annual crops and model plants generate a critical hypothesis for perennial systems (Table 2): in fruit trees, where root longevity and seasonal hormonal cycling create additional regulatory layers, IAA-based screening without considering edaphic context and phenological stage may be particularly prone to false positives. Yet no study has directly tested whether high-IAA PGPR strains inhibit root growth in established orchard trees, leaving this as an urgent validation need.

By stabilizing root metabolism, protecting membranes, sustaining antioxidant capacity, and reducing excessive ethylene accumulation, PGPR may help preserve plant vigor over time. However, this integrated framework still lacks long-term validation in multi-season orchard trials.

Unlike annual crops, fruit trees cannot rely on short life-cycle escape strategies; instead, they must tolerate repeated episodes of stress while maintaining long-term root viability, canopy integrity, reproductive development, and source-sink balance (Yang et al., 2026; Kesavardhini et al., 2025). By stabilizing root metabolism (Kesavardhini et al., 2025), protecting membranes (Haider et al., 2026), sustaining antioxidant capacity (Sobahan et al., 2025), and reducing excessive ethylene accumulation (Saraf et al., 2010), PGPR can preserve plant vigor over time and improve the resilience of orchard systems to recurring environmental disturbances. Therefore, the physiological effects of PGPR should not be interpreted as isolated plant responses independent of the surrounding ecological context.

As summarized in Figure 1, physiological stress alleviation is closely linked to the upstream rhizosphere processes described in Mechanism I and to the downstream signaling and defense priming discussed in Mechanism III. For example, improved rhizosphere water retention and ion buffering can reduce the initial intensity of salt or drought stress (Kesavardhini et al., 2025), while signaling molecules derived from PGPR can precondition host antioxidant and hormonal pathways before visible damage occurs (Mellidou et al., 2021).

Overall, physiological regulation of plant stress tolerance represents a central mechanism through which PGPR support sustainable fruit production.

### 2.3 Molecular signaling and induced systemic resistance

The third mechanistic layer involves molecular signaling and induced systemic resistance (ISR).

#### 2.3.1 Concept and signal perception of ISR

**Figure 4:**
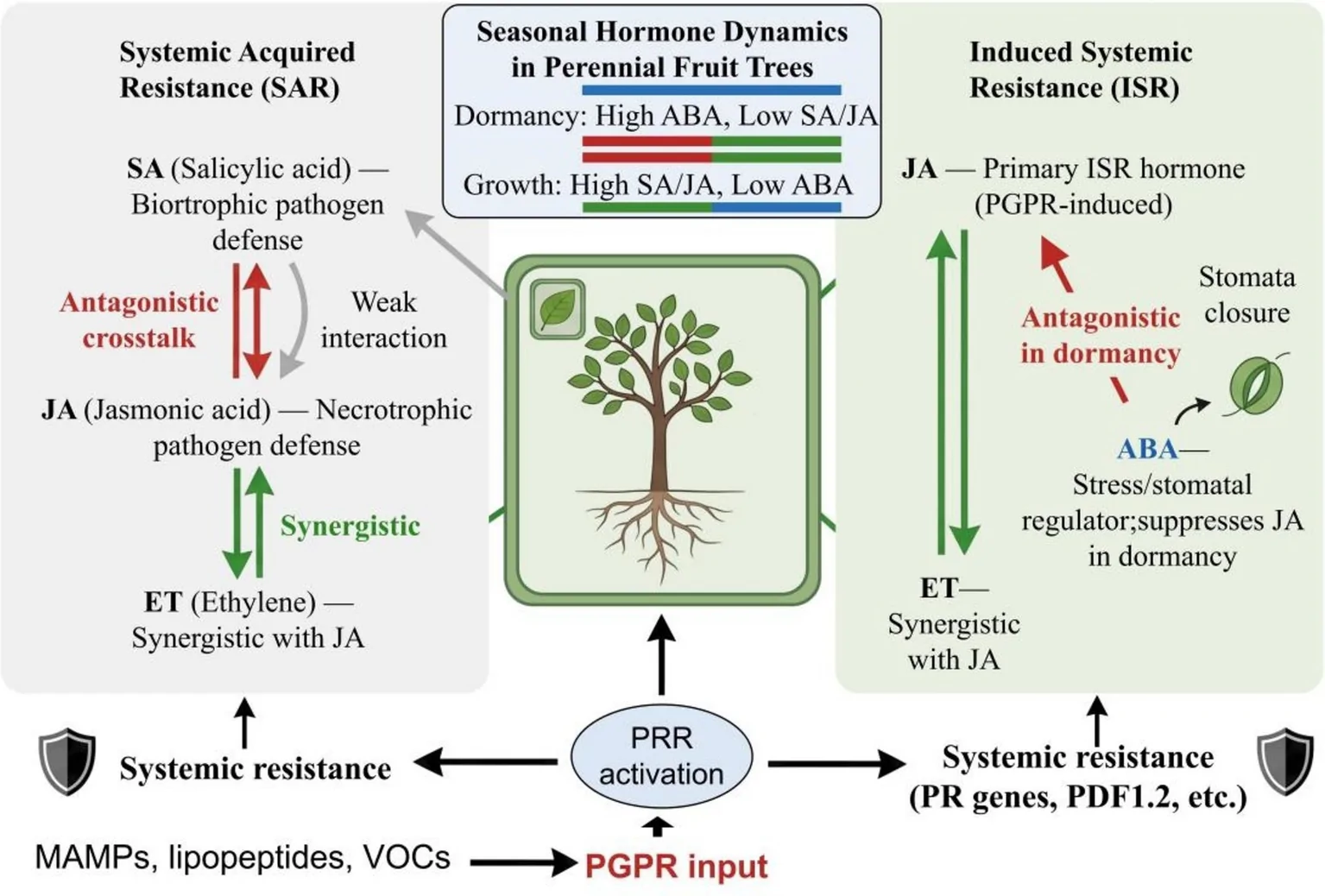
Hormone crosstalk in ISR.

The third mechanistic layer through which PGPR regulate fruit production systems involves molecular signaling and the induction of systemic resistance (Figure 4). In perennial fruit crops, this signaling function is particularly important because sustained productivity depends not only on immediate stress mitigation but also on the capacity to maintain a preconditioned, responsive defense state (Yuan et al., 2025; Muhammad et al., 2023).

In this context, PGPR-mediated stress regulation should not be viewed solely as a consequence of improved nutrition or reduced oxidative damage; it also involves the activation of immune-hormone-redox signaling before severe damage occurs (Yuan et al., 2025; Sibanyoni et al., 2026). This process is commonly referred to as ISR, in which root-associated beneficial microbes stimulate a primed physiological and molecular state that enables plants to respond more rapidly and effectively to subsequent stress challenges (Mellidou et al., 2021).

ISR is initiated when microbial cells or their metabolites are perceived by the plant as signaling cues (Mohammed et al., 2026). These cues may include microbe-associated molecular patterns (MAMPs), lipopeptides, volatile organic compounds, flagellin-derived peptides, siderophores, or other low-molecular-weight metabolites released during root colonization (Mohammed et al., 2026; Yasuda et al., 2025). Once recognized by pattern recognition receptors (PRRs) on the root surface, these microbial signals activate intracellular defense pathways involving Ca^2+^ influx, mitogen-activated protein kinase (MAPK) cascades, reactive oxygen species (ROS) bursts, and downstream transcriptional reprogramming (Yasuda et al., 2025). Through these early events, local perception in the root can be translated into systemic signaling outputs that affect distal tissues, including leaves, stems, and reproductive organs (Mashabela et al., 2022).

One representative example is the perception of the flagellin-derived peptide flg22, which has been widely used as a model MAMP to understand plant immune activation (Arteaga-Ojeda et al., 2026; Khan et al., 2023). This transferable evidence from model plants illustrates conserved receptor-mediated immune pathways that are also central to stress sensing in perennial species (Akinrinlola et al., 2018; Yang et al., 2024; Injamum-Ul-Hoque et al., 2024). However, direct validation of flg22-mediated PGPR perception in woody fruit crops remains limited, and whether signaling mechanisms identified in model plants operate similarly in orchard systems requires empirical testing.

#### 2.3.2 Hormone network integration and systemic coordination

A major feature of ISR is its close integration with plant hormone networks. As shown in Figure 4, unlike classical systemic acquired resistance (SAR), PGPR-induced ISR is commonly linked with JA, ET, and broader hormone crosstalk (Ho et al., 2026). Specifically, ISR is mediated through JA and ET pathways independent of SA, whereas SAR is associated with SA accumulation and PR proteins; this distinction enables plants to adopt distinct defense strategies against necrotrophic and biotrophic pathogens (Ho et al., 2026).

Furthermore, primed plants do not necessarily show strong defense outputs under non-stress conditions; instead, they remain in a sensitized state in which stress-responsive genes, antioxidant systems, and signaling modules can be activated faster and more intensely upon challenge (Mashabela et al., 2022). In the primed state, plants avoid constitutive activation of high-cost defenses; instead, they exhibit faster and stronger responses to minimal stimuli, representing a cost-effective form of induced immunity (Mashabela et al., 2022). This feature is particularly advantageous for fruit trees, because perennial species must repeatedly balance growth, reproduction, and defense over long life cycles (Rubby et al., 2025). A fully activated defense state may impose unnecessary energetic costs, whereas a primed state allows plants to maintain productivity while preserving the capacity for rapid stress adaptation (Rubby et al., 2025). PGPR-mediated ISR may therefore be especially valuable in orchard systems exposed to recurrent drought, salinity, high light, and pathogen pressure.

However, this theoretical advantage also introduces a risk specific to perennial systems: priming fatigue. Unlike annual crops that complete their life cycle within a single season, fruit trees must maintain responsive defense states across multiple years. Repeated seasonal activation of priming pathways—particularly under fluctuating environmental conditions—may lead to metabolic exhaustion or receptor desensitization if defense responses are not tightly regulated. This potential for priming fatigue in woody perennials remains largely unexplored and represents a critical gap in current understanding.

#### 2.3.3 Defense priming, molecular basis, and microbiome interactions

At the molecular level, ISR is characterized by defense priming rather than constitutive activation (Figure 4): an initial priming phase sensitizes defense genes without disturbing basal metabolism, and a later boost phase triggers rapid JA synthesis upon pathogen attack (Mathys et al., 2012). This two-phase model is derived from transferable evidence in Arabidopsis; direct evidence for PGPR-mediated priming in perennial fruit trees remains sparse. The molecular basis of this primed state includes transcriptional reprogramming, changes in secondary metabolism, and regulation of redox signaling (Yuan et al., 2025). Metabolomic analyses further reveal shifts in root and shoot metabolites involved in signaling, antioxidant responses, antimicrobial activity, and microbe-plant communication (Sibanyoni et al., 2026). These data suggest that PGPR do not merely activate isolated defense genes, but reshape broader regulatory networks that connect metabolism, signaling, and environmental responsiveness (Yuan et al., 2025; Muhammad et al., 2023; Sibanyoni et al., 2026). In fruit production systems, this systems-level regulation may help explain why some inoculants improve not only plant vigor but also resilience to complex, overlapping stress factors.

Unlike annual crops, fruit trees must sustain defense states across multiple years. In cherry seedlings, PGPR-mediated priming shows temporal partitioning: defense genes (CYP98A2-like, CYP73A100-like) upregulate at 40 days while growth markers decline (Tong et al., 2026). However, no study has tracked defense gene expression or hormone markers across multiple growing seasons in mature orchard trees. Transcriptomic snapshots at single time points cannot capture the cumulative, seasonally structured nature of PGPR effects in perennial systems. Whether this network rewiring persists, weakens, or fluctuates across dormancy cycles remains unknown.

Meanwhile, SA, JA, ET, ABA, and other signaling molecules interact dynamically to coordinate responses to pathogens, salinity, drought, heat, and oxidative stress (Pandey et al., 2025): SA and JA synergize through ET signaling, with ERF1 acting as a transcriptional integrator to activate both SAR and ISR; meanwhile, the SA-JA-ABA-ET network determines whether plants prioritize growth or long-term defense through exquisite balancing (Pandey et al., 2025). Hua et al. reported that Priestia megaterium 170 T-4 activated ethylene-related transcription factors and upregulated AOC (JA biosynthesis) while downregulating ACS genes in soybean (Hua et al., 2025). This transferable evidence illustrates microbial hormone reprogramming in an annual crop; direct validation in woody perennials—with their more complex phenological cycling and source-sink relationships—is lacking. Whether similar regulatory mechanisms operate in fruit crops with comparable magnitudes and temporal dynamics remains uncertain.

In addition to host signaling, PGPR-mediated ISR is often intertwined with microbiome-level interactions. Certain beneficial strains can release metabolites that recruit indigenous microbes, promote cooperative biofilm formation, or reshape community assembly in ways that reinforce defense-related functions (Zhang et al., 2022; Sun et al., 2022). For example, *Bacillus velezensis* SQR9 has been shown to promote enrichment of indigenous Pseudomonas spp. in the rhizosphere through metabolite-mediated recruitment, and these recruited bacteria can co-colonize roots and contribute to cooperative protection (Sun et al., 2022). Likewise, surfactin has been identified as a key determinant of biofilm formation and rhizosphere colonization in *B. velezensis*, in part through activation of the global regulator Spo0A (Zhang et al., 2022). These findings indicate that ISR should not be viewed only as a plant-internal process, but as an emergent outcome of signal exchange among roots, inoculated PGPR, and resident microbial communities.

#### 2.3.4 Multi-omics evidence and future perspectives

At present, much of the evidence supporting these signaling-based interpretations has come from multi-omics approaches. Traditional cultivation-based and physiological assays are often insufficient to resolve the complexity of PGPR–plant communication, whereas transcriptomics, metabolomics, and microbiome profiling have enabled the identification of signaling pathways, defense markers, microbial metabolites, and community-level responses associated with inoculation (Yuan et al., 2025; Sibanyoni et al., 2026). These methods have been particularly useful for linking PGPR treatment with host defense signaling, redox homeostasis, metabolic shifts, and rhizosphere community reassembly (Yuan et al., 2025; Muhammad et al., 2023). In this sense, omics technologies have not created the mechanism of ISR, but they have greatly improved our capacity to detect, interpret, and integrate its molecular components.

Molecular and multi-omics studies advance mechanistic understanding by identifying biomarkers of colonization and host response and by informing rational strain or consortium design. The integration of multi-omics evidence with field-based measurements of microbial persistence, host performance, fruit yield and quality, and stress outcomes across multiple seasons and environments represents a critical direction for future research. This integrated approach is needed to translate ISR-related mechanistic knowledge into reliable microbial strategies for perennial fruit production.

Despite these advances, omics-based interpretations of PGPR function remain subject to several important limitations. First, the majority of studies remain at the correlational level, rendering it difficult to identify key causal factors that directly drive plant trait improvement from gene expression, metabolite accumulation, or community alterations (Yuan et al., 2025; Muhammad et al., 2023). Transcriptomic shifts following PGPR inoculation may reflect direct microbial regulation, indirect host responses to improved nutritional status, or even stochastic variation unrelated to functional outcomes. Second, fruit tree-specific omics research remains relatively scarce compared with other horticultural species, thereby directly limiting the extrapolation of conclusions. Most transcriptomic and metabolomic evidence supporting PGPR-mediated ISR, hormone reprogramming, and antioxidant induction has been obtained from Arabidopsis, tomato, or soybean (Injamum-Ul-Hoque et al., 2024; Mathys et al., 2012; Hua et al., 2025). While the signaling logic is often conserved, perennial species present unique challenges: their longer life cycles require sustained rather than transient priming, and repeated seasonal activation may lead to priming fatigue or metabolic exhaustion if defense responses are not tightly regulated. Additionally, the hormone crosstalk central to ISR operates differently in woody plants, where ABA-mediated dormancy and phenological transitions interact with defense signaling in complex ways. Third, omics studies rarely integrate temporal dynamics with agronomic endpoints. A transcriptomic snapshot at one time point cannot capture the cumulative, seasonally structured nature of PGPR effects in perennial systems. Future omics research should therefore move beyond descriptive correlation and integrate multi-omics with long-term field tracking of colonization persistence, yield stability, and fruit quality across seasons.

## 3 Critical bottlenecks and translational challenges in orchard applications

**Figure 5:**
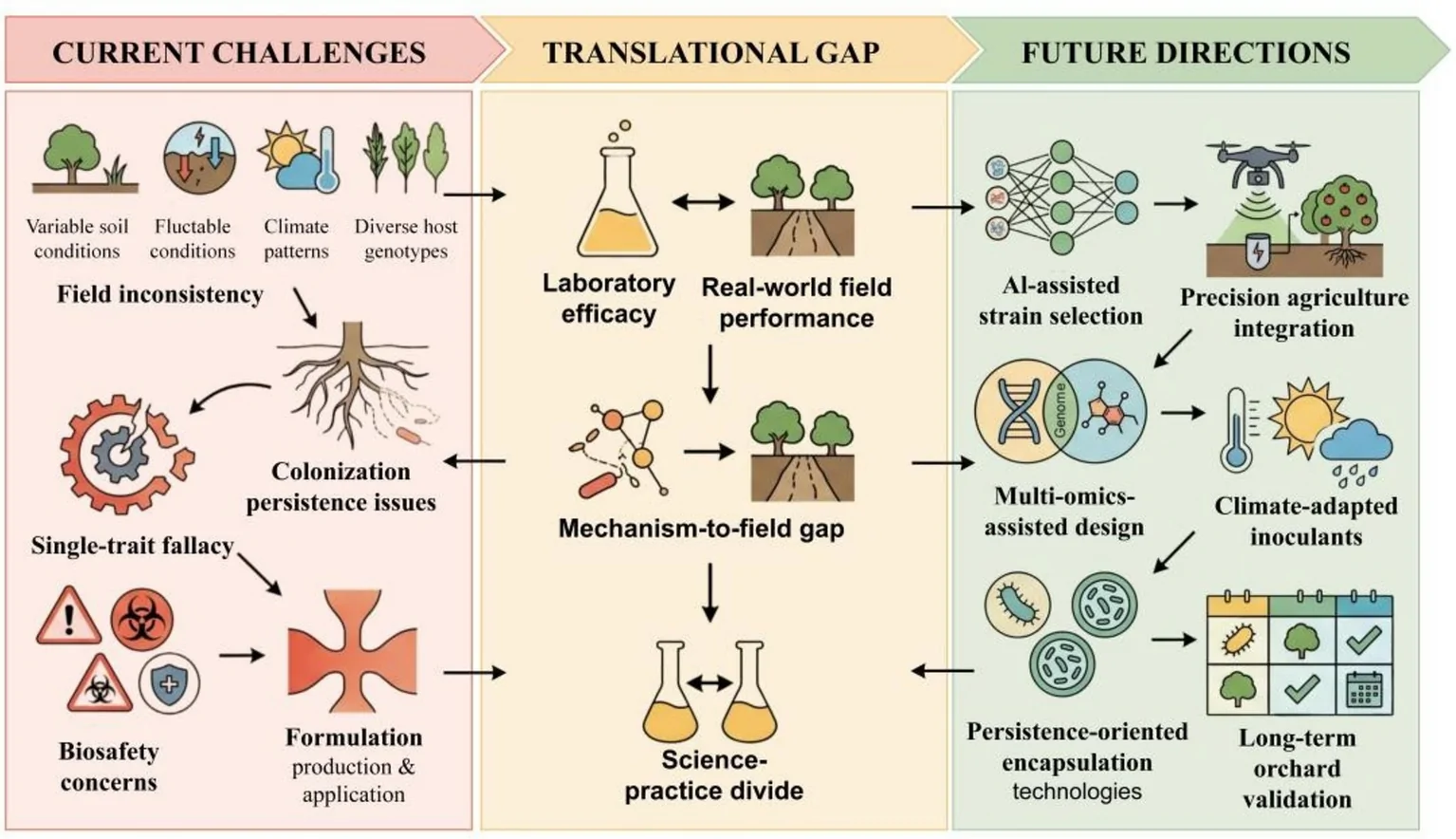
Translational challenges and future directions for PGPR in orchard systems.

Sections 2.1–2.3 show that PGPR regulate fruit production through interconnected ecological, physiological, and molecular pathways. However, mechanistic plausibility alone does not ensure reliable field performance. As summarized in Figure 5, the main bottlenecks include the limits of functional stacking in synthetic consortia, the single-trait fallacy in hormone-based screening, and the gap between correlation and causation in multi-omics interpretations. The following sections focus on these orchard-specific constraints and evidence gaps.

### 3.1 Field inconsistency: from laboratory efficacy to orchard reliability

Variation in PGPR efficacy among orchards reflects the strong context dependence of microbial activity, which is shaped by soil properties, climate, irrigation, fertilization history, host genotype, rootstock-scion combination, and interactions with resident microbiota (Zeng et al., 2025; Chiaranunt and White, 2023; Khan et al., 2022). Chiaranunt et al. explicitly noted that strains performing well under controlled conditions often show weak or short-lived effects in the field (Chiaranunt and White, 2023). In perennial systems, these sources of variation are compounded by long-term management practices and seasonal carryover effects that continuously reshape the rhizosphere environment. Field inconsistency should therefore be regarded as a central translational challenge. Progress will require host- and environment-specific inoculation strategies supported by multi-site, multi-season validation rather than short-term greenhouse performance alone.

Direct orchard evidence supports this concern. Ferreira et al. conducted a multiyear field evaluation of microbial biofertilizers in ‘Gala’ apple and found that treatment effects depended strongly on strain identity, rootstock, cultivar background, and evaluation criteria (Ferreira et al., 2026). Similarly, studies combining 16S rRNA sequencing, phenotypic screening, and pot inoculation in citrus have improved candidate PGPR identification (Xia et al., 2023; Li et al., 2025). Future studies should therefore prioritize ecological matching between inoculants and orchard conditions, while integrating field persistence, plant performance, and fruit production outcomes into the same evaluation framework.

### 3.2 Colonization persistence, ecological competition, and screening limitations

Establishment and long-term persistence are especially critical in orchard systems because many expected benefits, including stress buffering, physiological regulation, and induced systemic resistance, depend on sustained root association rather than transient exposure (Zhao et al., 2023; Fierro-Coronado et al., 2014). However, even strains with strong growth-promoting potential under controlled conditions often fail to establish durable populations after introduction into field soils (Santoyo et al., 2021).

Native microbial communities compete intensely for nutrients and root niches, while fluctuating temperature, moisture, salinity, soil structure, and management disturbance further reduce inoculant survival (Zhao et al., 2023; Santoyo et al., 2021). This persistence challenge is further complicated by interactions among inoculated strains themselves. Consortia composed of strains with substantial niche overlap may experience intensified resource competition, indicating that simply increasing inoculant diversity does not necessarily improve colonization success (He et al., 2025; Jia et al., 2026). In contrast, consortia with greater functional and ecological complementarity may achieve better persistence under competitive orchard conditions (He et al., 2025; Benmrid et al., 2025).

Future inoculant development should therefore prioritize persistence-oriented design. Strains should be selected for rhizosphere competence, host adaptation, ecological fitness, and compatibility with resident microbiota rather than solely for beneficial traits measured in simplified media. Formulation carriers, application timing, and delivery routes should also be optimized to improve establishment under orchard conditions, and long-term tracking of introduced strains across seasons should become a standard requirement in orchard PGPR evaluation.

### 3.3 The single-trait fallacy and mechanism-to-field gaps

The disconnect between laboratory screening and field performance reflects a deeper design flaw: single-trait screening—whether for IAA production, ACC deaminase activity, or antioxidant capacity—cannot capture the multi-factorial reality of orchard systems (Zhao et al., 2023; Fierro-Coronado et al., 2014). The IAA paradox and the unresolved validation of ACC deaminase in woody perennials exemplify how laboratory-screened traits may become irrelevant or even inhibitory under field conditions. Multi-trait, host-perception, and ecology-informed screening systems should therefore replace single-trait selection as the standard for inoculant development (Akinrinlola et al., 2018; Yang et al., 2024; Injamum-Ul-Hoque et al., 2024).

### 3.4 Biosafety, ecological risk, and unintended consequences

Biosafety and ecological risk, highlighted for selected taxa in Table 2 (⚠), must be treated as integral to inoculant design rather than peripheral afterthoughts. Strains with documented opportunistic pathogenic potential (e.g., *Pseudomonas aeruginosa*), toxigenic capability (e.g., certain *Bacillus cereus* genotypes), or poorly characterized long-term ecological effects should be excluded from agricultural recommendations unless strain-level safety data are explicitly provided (Mendybayeva et al., 2025; Sun et al., 2026).

Table 2 identifies these taxa for reference. For example, *S. maltophilia* BCM demonstrated favorable PGPR and biocontrol potential under diverse stress conditions, yet the authors explicitly cautioned that “long-term analyzes under field conditions are still required to validate these findings” before agricultural application (Sharma et al., 2024); *E. cloacae* has shown growth-promotion potential in maize and soybean, but its genome frequently harbors virulence factors and antibiotic resistance genes, with PathogenFinder scores indicating potential pathogenic risks (Amer and Hamed, 2025). Therefore, even non-pathogenic strains may exert profound influences on nutrient cycling, organic matter decomposition, and horizontal gene transfer through alterations in soil physicochemical properties or disruption of microbial interaction networks (Demyanyuk et al., 2025; Ahmad et al., 2018).

Therefore, biosafety evaluation should not be limited to short-term growth-promotion tests. Instead, routine screening should include: (i) strain-level taxonomic resolution, with exclusion of strains with known clinical or ecological risk; (ii) toxigenic and antibiotic resistance screening; (iii) assessment of potential long-term impacts on indigenous microbiomes; and (iv) risk–benefit assessment specific to perennial production contexts. Safe deployment is not separate from effective deployment; it is a prerequisite for credible orchard application.

### 3.5 Formulation constraints and the science–practice gap

Beyond biological and ecological constraints, limitations in formulation development and production control remain significant barriers to commercial application. The practical efficacy of PGPR products is determined not only by microbial functionality but also by cell viability maintenance, physiological activity preservation, strain equilibrium, storage stability, and effective field delivery capacity (Yang et al., 2024; Chompa et al., 2024). Gram-negative PGPR strains face severe challenges in bioformulation development due to their inability to form spores; microbial compatibility issues are particularly prominent, as many effective single PGPR strains are mutually incompatible in composite formulations (Ehinmitan et al., 2024). Storage stability studies have demonstrated that even spore-forming Bacillus strains exhibit shelf lives of only 6–12 months in talc-based carriers, with cell viability declining significantly over storage duration (Sah et al., 2011). At the field application level, artificial seed coating may reduce microbial viability and shorten the shelf life of coated seeds (Mousa et al., 2024). In the absence of reliable formulation technologies and quality control measures, potentially beneficial strains may become ineffective in orchard environments prior to expression of their beneficial functions.

A further disconnect exists between scientific outputs and market demands. Because some commercial inoculants can exhibit growth-promoting effects even without successful root colonization (Basu et al., 2021), a lack of standardized efficacy evaluation criteria has emerged, thereby compromising product reliability and farmer trust (Dutta et al., 2024). Numerous studies are driven by frontier pursuits that remain disconnected from field-level practical requirements, whereas enterprises encounter difficulties in articulating their technical needs with clarity, consequently leaving substantial research achievements underutilized (Zhang et al., 2025). This science–practice gap is particularly problematic for perennial fruit systems, where the long interval between inoculation and measurable yield outcomes makes empirical validation both essential and difficult to fund.

## 4 Precision integration and future directions

Taken together, these challenges indicate that the major obstacle in PGPR translation is not the lack of plausible mechanisms, but the gap between mechanistic understanding and field reliability. The next phase of research should therefore shift from proof-of-concept inoculation toward integrative translational design. More broadly, PGPR application in fruit production is likely to advance most effectively when integrated with precision orchard management and emerging technologies. This includes: (i) AI-assisted microbial selection, leveraging machine learning with microbiome profiling and environmental databases for predictive inoculant design (Zhang et al., 2025); (ii) precision agriculture-based microbial application, matching inoculation strategies with real-time soil diagnostics, climate risk modeling, and resident microbiome status (Chiaranunt and White, 2023; Rezaee Danesh, 2025; Tripathi et al., 2025; Qiu et al., 2019); (iii) digitalization of fruit growth and orchard microclimate monitoring, enabling data-driven optimization of inoculation timing and dosage; (iv) multi-omics-assisted strain selection, moving beyond single-trait screening toward host-perception and ecology-informed evaluation frameworks (Section 2.3.4); and (v) climate change-adapted orchard management, developing stress-preconditioned inoculants and drought-resilient consortium designs for changing environmental conditions. However, these technological advances must be grounded in long-term orchard validation; without multi-season, multi-site field trials, precision tools risk perpetuating the same laboratory-to-field gap that currently limits PGPR translation.

In summary, the future of PGPR in fruit production depends on moving beyond laboratory efficacy toward robust, safe, and context-dependent orchard deployment. If the mechanistic insights discussed in Section 2—and the critical limitations examined in this section—can be addressed through persistence-oriented design, formulation engineering, biosafety control, and long-term field validation, PGPR-based strategies may become a realistic component of sustainable orchard management rather than remaining primarily a promising experimental concept.

## 5 Conclusion

This review combines ecological, physiological, and molecular evidence to explain how PGPR regulate perennial fruit systems. Current evidence supports that PGPR improve orchards through the combined actions of roots, soil, and native microbes—not through isolated microbial traits alone. PGPR engineer the rhizosphere, stabilize plant physiology under stress, and trigger systemic resistance through molecular signaling. However, most studies still rely on annual crops, model plants, or short-term experiments. Fruit tree field trials remain scarce and inconsistent. Researchers have tested few strains under real orchard conditions, and results vary widely across sites and seasons. This unpredictability complicates the prediction of how inoculants persist and perform over multiple growing seasons. There is an urgent need for long-term, multi-site orchard experiments. These should track strain survival, yield stability, and fruit quality under real growing conditions. Researchers should also test synthetic microbiomes, combine multi-omics tools, and screen strains for ecological fitness in orchard soils. These steps will clarify why promising PGPR often fail in the field and will guide the design of better inoculants. Closer collaboration between microbiologists and horticulturists can bridge this gap. Finally, we must translate these findings into better formulations, practical delivery methods, and biosafe inoculation strategies. This will make PGPR applications more reliable and sustainable for fruit growers.
