# Correction: Emerging roles of PGPR in fruit production systems: from growth promotion to system-level regulation, a review

**DOI:** 10.3389/fmicb.2026.1966060

**Published:** 2026-08-25

**Authors:** Mengqi Zhao, Ming Li, Biao Zhang

**Affiliations:** 1 College of Viticulture and Enology, Ningxia University, Yinchuan, China; 2 Ningxia Institute of Grape and Wine, Yinchuan, China; 3 Engineering Research Center of Grape and Wine, Ministry of Education, Yinchuan, China; 4 College of Life Sciences, Northwest University, Xi'an, China

**Keywords:** fruit production systems, induced systemic resistance, PGPR, rhizosphere microbiome, system-level regulation

Author Mengqi Zhao was erroneously included as corresponding author.

The correct corresponding author is Ming Li.

The original version of this article has been updated.

